# Innovative Methods of Male Circumcision for HIV Prevention—Getting the Right Evidence

**DOI:** 10.1097/QAI.0000000000000738

**Published:** 2016-05-24

**Authors:** Julia Samuelson, Timothy Hargreave, Renee Ridzon, Tim Farley

**Affiliations:** * Key Populations and Innovative Prevention Team, Department of HIV and AIDS, World Health Organization, Geneva, Switzerland;; † Department of Clinical Sciences, Edinburgh University, Midlothian EH16 4TJ Scotland, United Kingdom;; ‡ Boston University School of Public Health, Boston, MA; and; § Sigma3 Services SÀRL, Nyon, Switzerland.

**Keywords:** male circumcision, medical devices, innovation research, implementation, prequalification, ShangRing, PrePex

## Abstract

World Health Organization recommends that countries with hyperendemic and generalized HIV epidemics implement voluntary medical male circumcision programs for HIV prevention. Innovative methods of male circumcision including devices have the potential to simplify the procedure, reduce time and cost, increase client acceptability, enhance safety, and expand the numbers of providers who may perform circumcision. We describe work led by World Health Organization and supported by global partners to define a pathway for the evaluation of efficacy and safety of male circumcision devices, to set priority criteria, and to establish a process to guide the use of devices in publicly funded voluntary medical male circumcision programs for HIV prevention. A device classification scheme, an expert Technical Advisory Group on Innovations in Male Circumcision, and a formal prequalification program have also guided considerations on safe use of devices. A rigorous approach was deemed appropriate given the intervention is for use among healthy men for public health purposes. The pathway and processes led to coordinated research, better standardization in research outcomes, and guidance that informed the research, introduction and implementation phases. The lessons learnt from this case study can inform evaluation and use of future public health innovations.

## INTRODUCTION

Based on compelling evidence,^1–5^ World Health Organization (WHO) and the Joint United Nations Programme on HIV/AIDS recommended in 2007 that countries with hyperendemic and generalized HIV epidemics implement voluntary medical male circumcision (VMMC) programs for HIV prevention.^6^ Modelling studies estimated that rapid scale up of VMMC would result in reduction of HIV incidence and be cost saving.^7^ Given the considerable requirements to implement and sustain this programme, simpler male circumcision methods that minimize surgical skills and requirements were and continue to be urgently needed. This article reviews work led by WHO and supported by global partners to define a pathway for the evaluation of efficacy and safety of innovative circumcision methods including devices, to set priority criteria, and to establish a process, including through the WHO Technical Advisory Group on Innovations in Male Circumcision (MC TAG), to guide the use of devices in publicly funded VMMC programs for HIV prevention. Lessons from this case study can be applied to future innovations that seek to address urgent public health needs.

### Device Landscape and Approaches to Fill Evidence Gaps

In 2008, potential technical innovations identified to simplify the male circumcision procedure included devices and innovations for anesthesia, hemostasis, and wound closure. Devices were assessed as the most promising approach to accelerate intervention delivery. The first systematic review on male circumcision devices^8^ found more than 20 commercially available products. Because few data, other than the claims by the inventors or manufacturers, were available on their clinical performance and safety for use in adult males, the MC TAG was established to advise on the minimum evidence needed and to review which devices could be considered for adult VMMC for HIV prevention in the priority African countries.

Given that low-resource countries have limited capacity to evaluate medical devices, WHO established in 2011 the Prequalification of Male Circumcision Devices Programme.^9^ WHO prequalification is a mechanism for guiding procurement decisions of Governments, United Nations, and other funding agencies. Through an objective and comprehensive assessment of a product dossier and inspection of the manufacturing site(s), the Programme ascertains whether a device has met international standards on design, testing, and adequacy of the quality management system. This body of information, together with evidence on clinical safety, performance and acceptability reviewed by the MC TAG, results in the decision to list a product as “prequalified.” These products are then considered suitable for public sector procurement. Prequalification does not imply approval or recommendation by WHO to use a specific device. Approval for device importation and use remains the prerogative and responsibility of national regulatory authorities and ministries of health. Information about the prequalification process and an up-to-date list of prequalified devices can be found on the WHO website.^10^ Prequalification is time-limited, regularly reassessed, and may be retracted if the manufacturer does not adhere to postmarketing surveillance requirements, maintain manufacturing quality controls, or if new concerns about safety arise.

### Which Evidence: WHO Clinical Evaluation Pathway

The MC TAG decisions on the evidence required were based on the following points:

- The risk–benefit balance of a prevention product is different than that for a therapeutic one. Male circumcision methods, including innovative devices, will be used on large numbers of healthy young men for the purpose of prevention.
- The assessment of efficacy, safety, clinical performance, and acceptability should be balanced with the importance of timeliness and practicality given the desired pace of scaling up VMMC as an effective HIV prevention intervention.
- Circumcision devices must be objectively evaluated before they are used in public health VMMC programs with evidence from at least 2 randomized controlled comparisons and 2 field research studies in at least 2 different settings or countries.

Evaluation issues center on clinical performance, efficacy, safety, ease of use, acceptability, and feasibility, including cost. Specific criteria to evaluate these aspects of an innovative device method include:

- Perspectives of clients (eg, pain control, healing and associated abstinence time, interference with normal activities)
- Perspectives of providers (eg, ease of use, procedure time, reproducibility, safety)
- Safety (eg, hemostasis, protection of glans, adverse events, minimizing risk of cross infection, including through single-use devices)
- Program considerations (eg, cost, training needs, supply management).

A preliminary evaluation pathway was developed in 2009 by experts in medical device regulation, research, public health program delivery, and clinicians with experience in resource-limited settings. The MC TAG members further advised, developed, and adopted in 2012 the *Framework for Clinical Evaluation of Devices for Male Circumcision*.^11^ The *Framework* serves as the basis for a uniform approach to research and assessment of circumcision devices and other innovations and describes steps to progress from an initial series of studies to phased implementation in specific national contexts. Because the purpose of using a device for VMMC was to develop a standardized method that simplified the procedure and reduced riskier steps, such as injected anesthesia, incision, and sutures, studies with diverse health care cadres in more than 1 geographic location were deemed necessary.

The first step on the pathway is to establish the clinical profile through review of available clinical data and experience in the country of origin or manufacture and assess its relevance to the population and setting of intended final use. If deemed promising, the device should be evaluated in a series of studies in countries or settings of intended final use, including case series, randomized comparative studies by skilled surgeons, studies on acceptability by providers and clients, and field studies involving trained mid-level clinical providers to demonstrate their potential to perform circumcision safely (Table 1). In addition to the clinical studies, phased implementation is advised beginning with country-led pilots and adverse event surveillance that would inform progressive expansion (Table 2).^12^

**TABLE 1.**
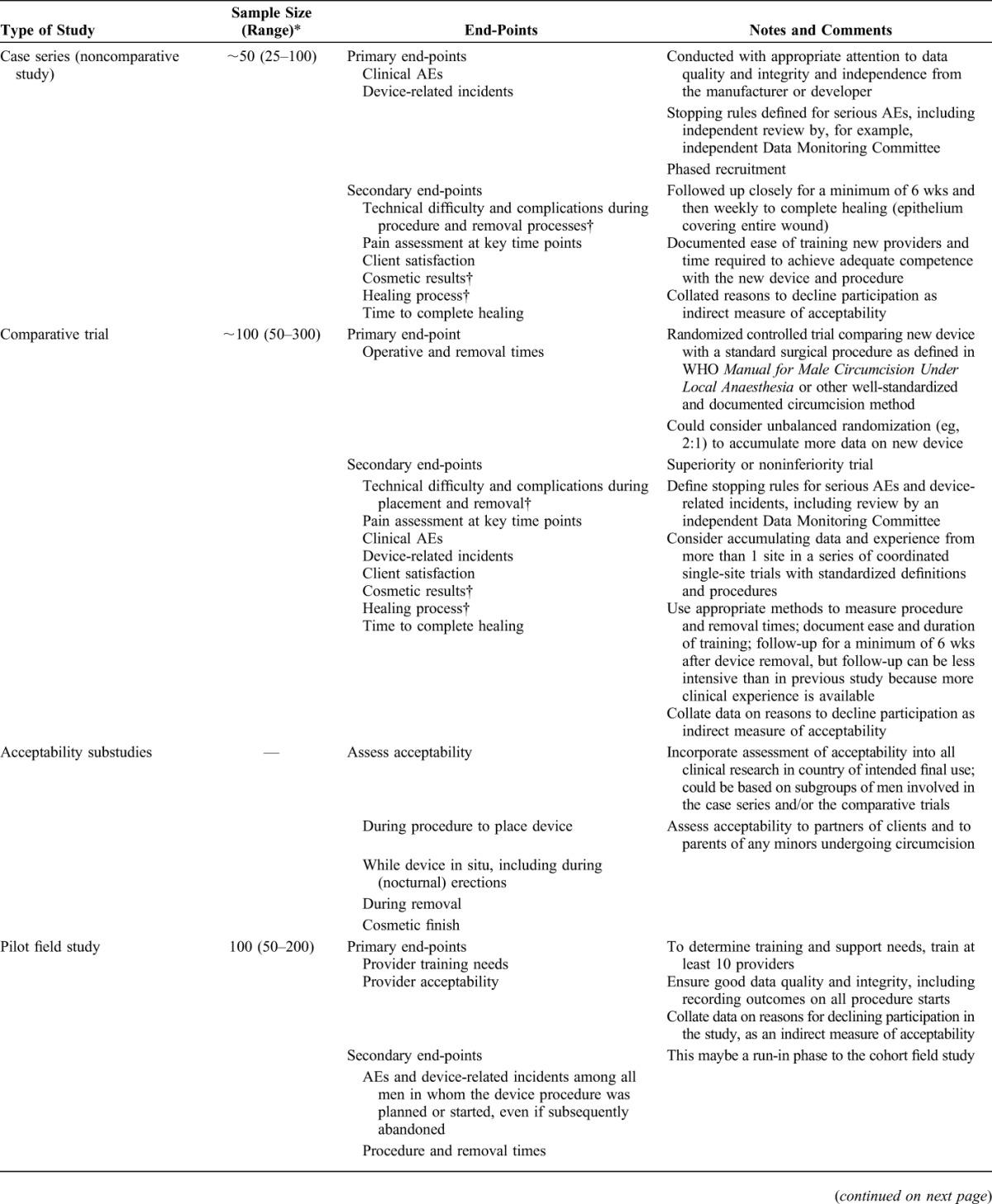

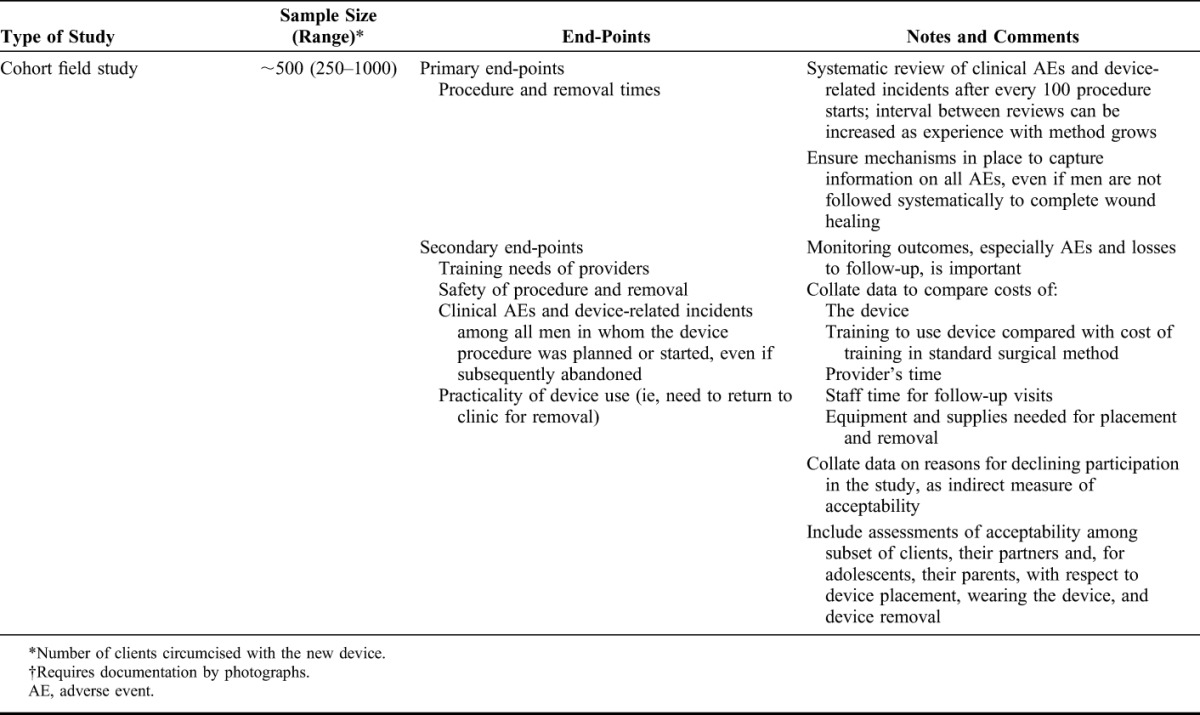
Research Pathway for Clinical Evaluation of Innovative Male Circumcision Methods: Clinical and Field Studies in Country or Setting of Intended Final Use

**TABLE 2.**
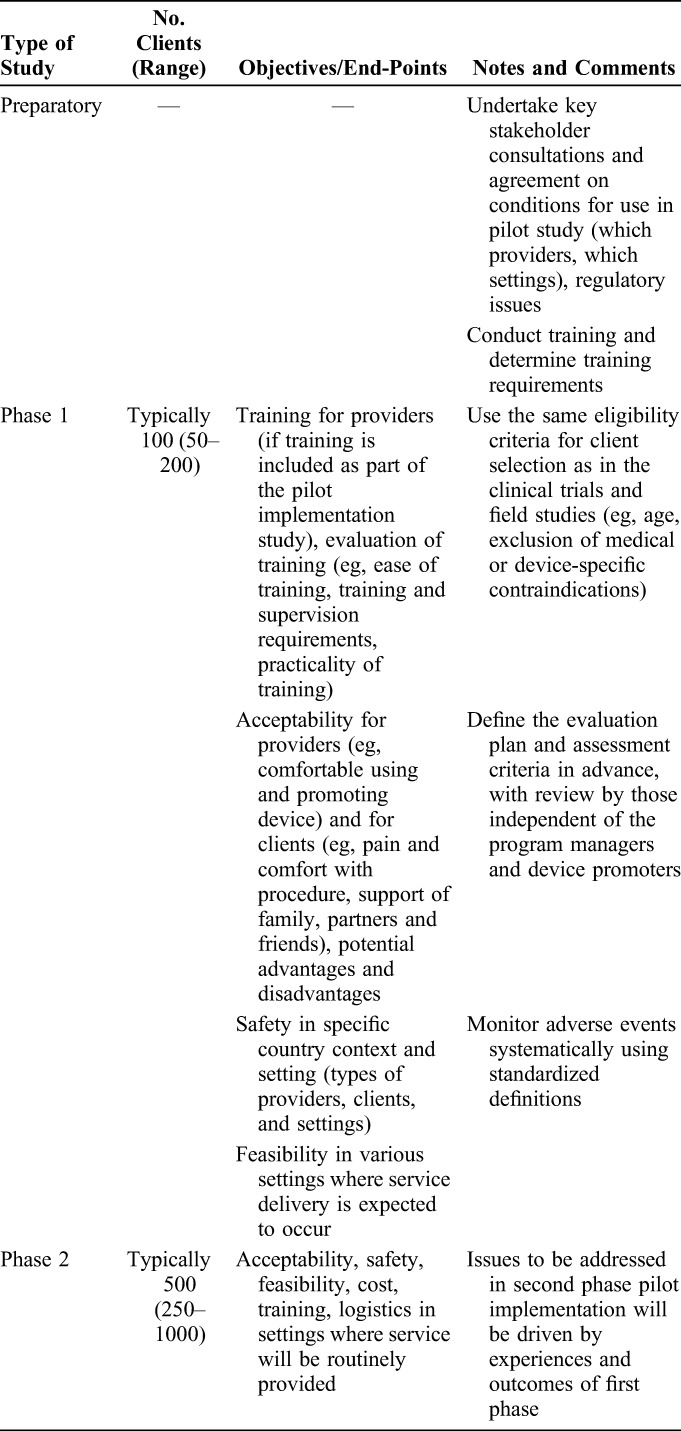
Pilot Implementation Studies: One Step in Introducing a New Male Circumcision Method

Although 2 comparative and 2 field studies are required for prequalification (see below), the total number of clients in these studies (minimum 1200) gives limited statistical power to show rare but potentially serious adverse events. Following the initial studies that inform prequalification of a device, active adverse event surveillance should document outcomes among at least the first 1000 clients within each country. If the complication rates are within acceptable limits, broader scale-up with passive surveillance should continue, documenting adverse events among clients who return.

The MC TAG advised that initial studies be conducted on men aged 18 years and older in accordance with local regulations on age of research participants. Once safety and acceptability had been established in adult populations, bridging studies involving at least 200 clients were necessary to demonstrate clinical safety in younger age groups.

### Classification of Devices

To provide a framework for considering issues and potential risks with different types of circumcision devices, the MC TAG developed a classification system for male circumcision devices according to the time that a device remains on the body and the mechanism of action (Table 3). This comprehensive classification covers male circumcision devices relevant for infants, adolescents, and adults. Three device categories by mechanism of action are (1) clamp, (2) elastic collar compression, and (3) ligature compression. Clamp devices deliver a rapid, tight compression of the foreskin sufficient to achieve hemostasis and prevent tissue slippage such that the foreskin can be removed at the time of device application; injected local anesthesia is required. Part or all of device is left in situ, usually up to 1 week. The elastic collar compression-type device provides slow compression between an elastic ring and a hard inner ring sufficient to cause ischemic necrosis of the foreskin over a period of 1 week while the device remains in place. Ligature compression devices are similar except that a nonrigid ligature holds the foreskin in place against an inner hard ring. These different mechanisms of action have unique risks that inform monitoring.

**TABLE 3.**
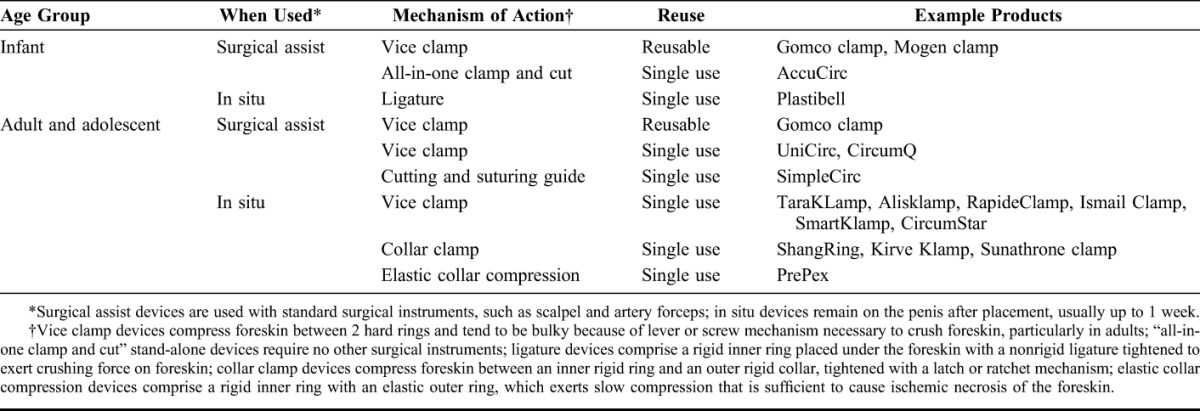
Categories of Medical Male Circumcision Devices With Examples

### Getting the Evidence: Application of the Framework

Two innovative devices that are currently prequalified, the PrePex and ShangRing, are used as examples of the application of the *Framework*. The PrePex is considered simpler than surgery as no local injectable anesthesia or suturing are needed as the foreskin is removed at a second visit after 1 week. The recommended studies were conducted in 3 countries with support from ministries of health, President's Emergency Plan for AIDS Relief, the Bill & Melinda Gates Foundation, and the United Nations Population Fund. The first-in-human study involving 55 men was conducted in Rwanda in 2010.^13^ A randomized comparison between the device and surgery in 217 men demonstrated that the device resulted in successful circumcision but could not be placed on about 10% of men because of phimosis or narrow foreskin opening.^14^ A subsequent pilot study with mid-level providers in 49 men^15^ and a field study in 590 men^16^ demonstrated safety and acceptability. These data were reviewed in 2012 by the MC TAG, which advised collection of further evidence in a different country and setting.^17^

In January 2013, results from 3 studies in Zimbabwe were reviewed^18^—an initial study with 53 men, a randomized controlled trial in 240 men, a field study using mid-level providers in 642 men, and 2 field studies from Uganda involving a total of 921 men.^19,20^ In total, more than 2500 men aged 18 years and older were enrolled in these studies. Approximately 7% of men could not have the PrePex device placed for various anatomical and technical reasons. Adverse events occurred in 1.7% of men, most of which were mild or moderate. Serious adverse events occurred in 0.4% of men and more than half were because of device displacement or removal (including self-removal). These displacements resulted in pain, swelling, and occasional blistering of the partially necrotic foreskin, requiring rapid intervention by a skilled surgeon to prevent severe local or systemic infection and/or permanent sequelae. MC TAG concluded that the device was efficacious and safe among healthy men aged 18 years and older when used by trained mid-level providers in public health programs. It advised that skilled surgical backup be available within 6–12 hours to manage complications, particularly displacements, and that conventional surgical circumcision needed to be available to the 10%–15% of adult men who could not be circumcised by PrePex.

Other components of the WHO prequalification process (review of product dossiers, assessment of manufacturing quality management systems) were undertaken in parallel with the clinical evaluation. PrePex was prequalified by WHO in 2013 for use in men aged 18 years and older, allowing its use within national programs. Most national programs, supported by key global partners, have undertaken the phased approach to implement this new method.

The ShangRing does not require suturing but requires injected local anesthesia and a return visit after 1 week for device removal. The foreskin is removed at the time of placement. The device had been initially assessed in multiple research studies in China.^21^ For the review of ShangRing use in African settings, evidence from men aged 18 years and older was available from Kenya, Zambia, and Uganda. It consisted of an initial case series (40 men),^22^ a study of spontaneous detachment (50 men),^23^ a randomized comparison with surgery (400 men, 200 allocated to ShangRing),^24^ and 3 field studies (total 1764 men).^25,26^ Approximately 1% of men were ineligible for the ShangRing because of foreskin abnormalities. During device placement, 0.4% men (7) required rapid intervention with surgical circumcision as the excision had occurred but the foreskin slipped from the device and required suturing. No serious adverse events occurred; 20 men (1.0%) experienced moderate adverse events from a total of 1983 successful device placements. All adverse events were managed with minor interventions and resolved without long-term sequelae. Rates were similar to those observed with conventional surgical circumcision. The MC TAG concluded that the ShangRing was efficacious and safe for use in healthy men aged 18 years and older when performed by trained providers in public health programs and that skilled surgical backup needed to be available to convert device placement failures to a conventional procedure.^27^

In September 2014, additional evidence which met the *Framework's* minimum data requirements on the clinical profile of the ShangRing from studies in Kenya and Uganda in 357 adolescents aged 13–17 years was reviewed and showed the device to be safe and acceptable in this age group.^28,29^ In June 2015, the ShangRing manufacturer satisfactorily met the necessary prequalification requirements and the device was prequalified for males 13 years and older.

In a bridging study among adolescents aged 13–17 years in Zimbabwe, PrePex could not be placed in 36% because of a narrow foreskin opening or adhesions between glans and foreskin. However, the device was safe and acceptable in the 402 successful placements.^30^

Because of limited numbers of device (PrePex and ShangRing) placements in those 13–17 years, MC TAG advised active surveillance of 2000 adolescents from at least 3 countries for each specific device. This surveillance will provide further evidence on safety and use.

The MC TAG also reviewed data on more than 24,000 PrePex device placements from 14 pilot implementation studies in 10 countries [Botswana, Kenya, Lesotho, Malawi, Mozambique, South Africa (3), Uganda (3), United Republic of Tanzania, Zambia, Zimbabwe], 6 active surveillance studies [Botswana, Rwanda, South Africa, Uganda (2), Zimbabwe], and passive surveillance in Rwanda and Zimbabwe. Safety in the hands of mid-level providers was consistent with earlier reports and displacements occurred at a similar rate; all were successfully managed by prompt referral to skilled surgery. A small number of tetanus cases after both conventional surgical and PrePex circumcision were reported from routine surveillance of VMMC programs. In response, an expert consultation was convened by WHO^27^ to further review and advise on tetanus risk and mitigation with surgery and device use. Overall, the monitoring during phased implementation provided further evidence to guide use and safety.

## DISCUSSION

**FIGURE 1.**
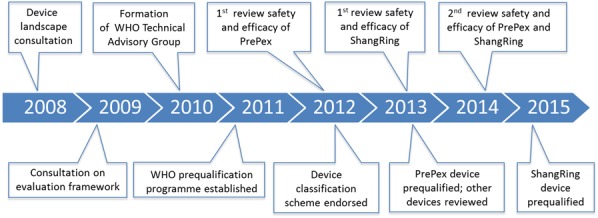
Timeline for WHO technical reviews and prequalification process on male circumcision devices.

The *Framework for Clinical Evaluation of Devices for Male Circumcision* has been instrumental in facilitating uniform and relevant research to evaluate an innovative method. The clear research pathway, objective evaluation criteria, and a rational sequence for phased implementation are likely relevant to other public health innovations. Although the process from innovation to research and expanded use seems long and cumbersome (Fig. 1), all the steps, without shortcuts, remain necessary to ensure safety, acceptability, and suitability for use in public health programs. In addition for prequalification, manufacturing procedures and documentation considered adequate to support quality large-scale production take time to develop, implement, and test. Although the clinical requirements defined in the *Framework* are substantially more stringent than those normally required by national regulatory authorities for circumcision devices, they are appropriate given the risk–benefit balance of a medical device used for prevention purposes.

A number of advantages and lessons emerged through this process (Table 4). First, countries and funders have unbiased information on circumcision devices suitable for use within publicly funded VMMC programs. The prequalification process does not replace national regulatory authority responsibilities to authorize and oversee marketing of medical devices but provides them with additional information on which to base decisions. Second, from the perspective of the device manufacturer and stakeholders providing technical or funding support, a clear evaluation pathway provides clarity on the assessments and evidence needed. Third, the process of developing the *Framework*, evaluation of devices, convening of the MC TAG, and prequalification activities required input from multiple stakeholders in the form of personnel time and financial resources. The support that came primarily from Bill & Melinda Gates Foundation and President's Emergency Plan for AIDS Relief was considerable and likely exceeded the manufacturers' contributions to development costs of the devices. This external support reinforces the need for fair public sector pricing of devices (Table 5).

**TABLE 4.**
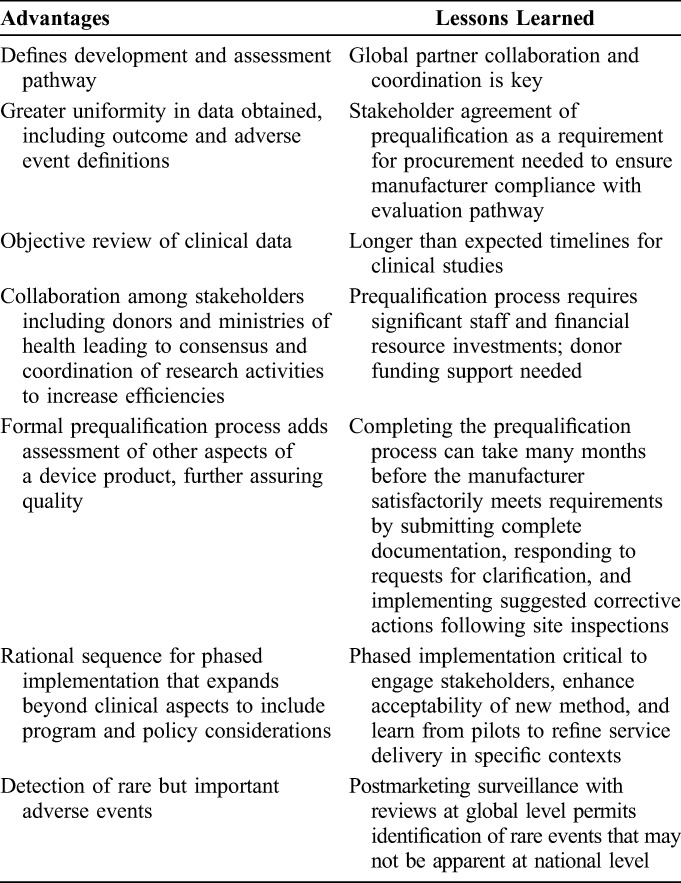
Advantages and Lessons Learned on Development and Implementation of Evaluation Criteria for Male Circumcision Devices

**TABLE 5.**
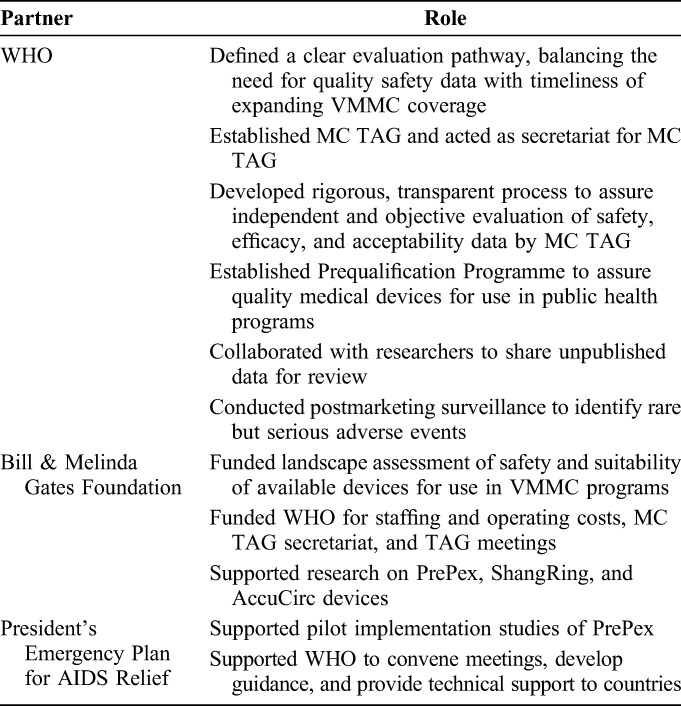
Roles of Principal Collaborating Partners

Initial device studies were undertaken on adult men. Countries and implementing partners have queried the safety and effectiveness of specific devices for adolescents and infants. Safe methods for these age groups are becoming increasingly important as countries consider how to sustain high prevalence of VMMC and reduced risk of HIV infection once 80% of men aged 15–49 years are reached. The pathway described in the *Framework* also applies to the assessment of novel infant and adolescent male circumcision devices.

The processes described to evaluate safety with innovative methods are relevant for other global public health interventions supported through limited international and domestic financial resources. Lessons that have been learned to date and that will continue to accrue should be used to inform introduction and broad implementation of other public health interventions.
